# Dengue epidemic in Malaysia: Not a predominantly urban disease anymore

**DOI:** 10.1186/1756-0500-4-216

**Published:** 2011-06-29

**Authors:** Nor Azila Muhammad Azami, Sharifah Azura Salleh, Hui-min Neoh, Syed Zulkifli Syed Zakaria, Rahman Jamal

**Affiliations:** 1 UKM Medical Molecular Biology Institute, Universiti Kebangsaan Malaysia, Malaysia; 2 Department of Medical Microbiology and Immunology, Universiti Kebangsaan Malaysia; 3 Secretariat, The Malaysian Cohort, Malaysia

## Abstract

**Background:**

Dengue infection has been an important and serious public health concern in Malaysia ever since its first reported case here in 1902. Nevertheless, to our knowledge, no nationwide investigation has been carried out to determine the actual magnitude of dengue endemicity in the Malaysian population. In this study, we describe a cross sectional seroepidemiology study of dengue IgG seroprevalence in the Malaysian adult population.

**Findings:**

From 1000 subjects (35-74 years old), 91.6% subjects were found to be dengue seropositive. Age is found to be a significant risk factor associated with dengue seroposivity, where the seroprevalence increased with every 10 year increase in age. Nevertheless, gender and ethnicity did not have an effect. Interestingly, there were similar seroprevalence rates between urban and rural samples, showing that dengue is presently not confined to urban areas in Malaysia.

**Conclusions:**

High dengue IgG seropositivity found in the population is an indication that dengue might be endemic in Malaysia for a long time into the future. Public awareness, proper vector control and vigilant surveillance are critical to keep the infection rates low and to prevent outbreaks.

## Findings

### Background

Dengue infection has been an important public health concern in Malaysia ever since its first reported case here in 1902 [1]. The virus (DENV) is transmitted by the mosquito *Aedes aegypti*, which is usually found in urban areas [2]. Across the world, about 100 million cases of dengue infection and 500 000 cases of dengue haemorrhagic fever (DHF) with 12 000 deaths were reported per year [3].

Clinical manifestations of dengue infection ranges from non-specific febrile illness to classical dengue fever (DF), DHF and dengue shock syndrome (DSS) [4]. Reported cases that are accounted for by public health statistics are usually secondary infections, where symptoms of DF, DHF or DSS might be present. These clinical manifestations are all thought to be a consequence of a heightened immune response due to cross-reactive T-cell responses and/or enhancing dengue antibody [5-7]. On the other hand, first infection by DENV is usually subclinical and can present atypically as an undifferentiated febrile illness [8].

Malaysia, with a population of approximately 27.7 million and a population density of 84 per sq. km, has continuously recorded rising annual cases of dengue infection since 1980. Major, national dengue outbreaks were reported in 1974, 1978, 1982 and 1990, exhibiting a 4-year cycle [9]. According to the 2008 Health Facts by Malaysian Ministry of Health, the incidence rate of dengue was 167.76 per 100 000 population with a mortality rate of 0.02 [10]. In the process of becoming a developed nation, massive infrastructure development in this country has contributed towards a high incidence of dengue infection, as urbanization is a favourable factor for *Ae. aegypti *breeding and subsequently facilitates the spread of DENV [11-13].

As primary exposure to the virus is usually asymptomatic, the actual magnitude of dengue infection in Malaysia may be larger than expected. We hereby conducted a cross-sectional seroepidemiology study to determine the prevalence of dengue antibodies in the Malaysian adult population. This study will be an initial step in estimating the extent of the dengue epidemic in Malaysia.

#### Study population

The Malaysian Cohort (TMC) is a national project managed by our research institute (UKM Medical Molecular Biology Institute, UMBI). Initiated in 2006, this nation-wide cohort recruits Malaysians aged 35 and above; its participants are considered as the best representative of the Malaysian adult population as they represent subjects from various ethnic groups, geographical locations and lifestyles. Detailed information about each participant is collected along with blood, serum, plasma, lymphocytes and urine samples. All subjects were healthy during the time of sample collection and consented towards the storage and usage of their samples for medical and epidemiological research. Ethical approval for The Malaysian Cohort has been granted by the Universiti Kebangsaan Malaysia Research Ethics committee.

There were a total of 13725 participants recruited into the TMC from 1 January 2008 until 31 December 2008. We randomly selected 1000 subjects from the initial population of 13725 using Minitab 15 software (Minitab Inc., Pennsylvania, USA), and retrieved their serum samples for our study. Socio-demographic data of the subjects such as gender, age, ethnicity, locality (urban/rural) were also retrieved from the TMC database (Table 1).

**Table 1:** Sociodemographic data of 1000 subjects whose serum samples were used in this study

Sociodemographic factor	Number of subjects*	**Dengue IgG positive**^**¥**^	**Dengue Seroprevalence**^**£**^
**Gender**			
Male	400 (40.0%)	375	93.75
Female	600 (60.0%)	541	90.17

**Age Range**			
35-44	137 (13.7%)	110	80.29
45-54	474 (47.4%)	439	92.62
55-64	338 (33.8%)	319	94.38
65-74	51 (5.1%)	48	94.12

**Median Age, y (Range)**	53 (35-74)		

Ethnicity			
Malay	570 (57.0%)	523	91.75
Chinese	371 (37.1%)	339	91.37
Indian & Others	59 (5.9%)	54	91.53

**Locality**			
Rural	398 (39.8%)	362	90.95
Urban	602 (60.2%)	554	92.03

*Except where indicated, values are (n, %)

^¥ ^Except where indicated, values are (n)

^£ ^Except where indicated, values are (%)

#### Dengue IgG antibody detection

Serum samples were measured for the presence of dengue virus-specific IgG antibodies using the PanBio Dengue IgG INDIRECT enzyme-linked immunosorbent assay (ELISA) (Inverness Medical Innovations Australia Pty Ltd, Queensland, Australia). This kit identifies antibodies to all four DENV serotypes but cannot differentiate between each serotype. Positive result of dengue IgG antibody indicates previous exposure to DENV. Interpretation of ELISA results were made according to instructions from the manufacturer.

#### Data Analysis

Data analysis was performed using Statistical Package for Social Science (SPSS) 16.0 (SPSS Inc., Chicago, USA). Chi square analysis was used to determine the relationship between genders, age groups, ethnicity and locality with dengue IgG seroprevalence. Logistic regression analysis was used to determine risk factors associated with positive dengue IgG. Odd ratios and their 95% confidence intervals were provided as estimates of the effect size and the chi-square tests were conducted at 3% level of significance while logistic regression tests were conducted at 5% level of significance.

## Result and Discussion

From the 1000 subjects surveyed, 916 (91.60%) were positive for dengue IgG, where 541 (90.17%) females and 375 (93.75%) males were dengue seropositive. This shows that a large proportion of the Malaysian adult population has been exposed to DENV. Ethnically, dengue IgG seroprevalence were as follows: Malay, 91.75% (523/570); Chinese, 91.37% (339/371); Indian and others, 91.53% (54/59) (Note: In 2008, the ethnicity ratio of recruited subjects in the TMC was comparable to the national ratio of Malay: Chinese: Indian = 6:3:1). Chi square analysis showed no significant difference in dengue IgG seroprevalence between different ethnic groups (χ^2 ^= 0.43, p = 0.979) and gender (χ^2 ^= 4.055, p = 0.045); the seroprevalence rates were more than 90% for each category for both demographic factors (gender and ethnicity). This shows that a large proportion of the Malaysian adult population has been exposed to DENV; there were neither gender bias nor ethnicity preferences.

Nevertheless, we noticed that dengue IgG seroprevalence rates increased with age among our subjects, and the difference between age groups was significant (p < 0.001) (Table 1). The odds of dengue seropositivity (compared to the 35-44 years old age group) were as follows: 45-54 years old, 3.08 (95% CI: 1.79-5.31); 55-64 years old, 3.89 (95% CI: 2.07-7.30) (p < 0.001); and 65-74 years old, 3.59 (95% CI: 1.03-12.46) (p = 0.045) (Table 2). This finding is in conjunction with reports that dengue seroprevalence will increase for every 10 year increment in age; older subjects will have a higher chance of infection [4,14]. As the TMC only recruits adults 35 years old and above, dengue seroprevalence in the younger population is not known; it remains to be investigated if there was a sharp rise in the seroconversion rate during childhood/young adulthood, or whether the conversion has occurred gradually.

**Table 2:** Univariate analysis of risk factor for dengue IgG seropositivity

Covariates	Odd Ratio (OR)	95% Confidence Interval (CI)	**P-value**^**§**^
Age Range			
35-44	1.00	(1.79, 5.31)	**< 0.001**
45-54	3.08	(2.07, 7.30)	**< 0.001**
55-64	3.89	(1.03, 12.46)	**0.045**
65-74	3.59		

**Bold**^**§ **^indicates significant values, where p < 0.05

Surprisingly, there was no difference (p = 0.550) in the dengue IgG seroprevalence between subjects staying in urban areas (92.03%, 554/602) compared to those living in rural communities (90.95%, 362/398), even though dengue is usually described as an "urban" disease [15]. In our study, samples from "urban" areas were retrieved from the TMC control centre (based in our university hospital, UKM Medical Centre in Kuala Lumpur, Malaysia) and also town areas; while samples categorized as "rural" were collected from subjects living in villages and also settlers in palm oil plantations from the Federal Land Development Authority (FELDA) communities nationwide. Hayes et al. (1990) reported that dengue antibody seroprevalence was high in urban (66%) compare to rural populations (26%) or jungle areas (32-64%) [16]. Population-based study in rural Amazonia of Brazil also showed a lower baseline DENV seropositivity rate of 18.3% [17]. Nevertheless, our study showed that there was no difference in the seroprevalence between urban and rural areas. This phenomenon might be caused by rising vector populations in rural areas. A study by Chen et al. in 2005 gave an ovitrap index of 56% in a rural settlement [18]. However, in a separate study carried out 2 years later in two insular rural settlements, the ovitrap index was 63% and 80%, respectively [19]. In addition to rising vector populations, improved modes of transportation which allow migration of viramic subjects from one place to another might have facilitated the spread of dengue from urban into rural areas.

The high dengue IgG seropositivity found in the Malaysian adult population (91.6%) indicates that 9 out of 10 adults are already exposed to dengue infection. This percentage of dengue IgG prevalence is higher compared to that among adults in Singapore (45%) and Central Brazil (29.5%) based on house-hold surveys, but lower compared to the study among adults in Santo Domingo, Dominican Republic (98%) [4,11,14]. The major drivers for high dengue IgG seroprevalence in Malaysia are probably demographic changes, massive urbanization in town areas, high density of vector populations such as *Ae. aegypti *in both urban and rural areas and changes in the usage of agricultural land in rural locations which promotes the propagation of *Aedes sp *[11,18]. Lack of effective mosquito control programs may have also helped to spread the virus.

In this study, the IgG detected was considered to be specific for DENV, even though the ELISA kit used might also detect other flavivirus antibodies such as JE and Yellow Fever (YF). In Malaysia, DENV and JE viruses are two of the more common endemic flaviviruses; YF and West Nile viruses have never been isolated here. Compared to dengue, the number of JE cases in Malaysia is minimal (annual incidence of JE virus infection is 9.8 per 100 000 population compared to 167.76 per 100 000 population for dengue [10,20]. Other flaviviruses found in Malaysia such as Tembusu and Langat do not cross-react with dengue antibodies [21]. Taking it all together, our positive ELISA results were more likely to be caused by dengue IgG than antibodies from other flaviviruses.

## Conclusion

To the best of our knowledge, this is the first dengue IgG seroprevalence study carried out on healthy adults in Malaysia. High dengue IgG seropositivity (91.60%) found in the population is an indication that dengue might be endemic in Malaysia for a long time into the future. Public awareness, proper vector control and vigilant surveillance are critical to keep the infection rates down and to prevent outbreaks. It remains to be determined which dengue serotype is more dominant, and if the dengue serotypes found in this population are similar to or different from the current circulating serotypes in the vectors.

## Competing interests

The authors declare that they have no competing interests.

## Authors' contributions

AA carried out the immunoassay, performed statistical analysis and drafted the manuscript. SA participated in the design of the study and data interpretation. HM participated in the design of the study and drafted the manuscript together with AA. SZ participated in the design of the study and coordination. RJ conceived the study and participated in the study design and coordination. All authors read and approved the final manuscript.
